# The Diversity of Prokaryotic DDE Transposases of the Mutator Superfamily, Insertion Specificity, and Association with Conjugation Machineries

**DOI:** 10.1093/gbe/evu010

**Published:** 2014-01-13

**Authors:** Romain Guérillot, Patricia Siguier, Edith Gourbeyre, Michael Chandler, Philippe Glaser

**Affiliations:** ^1^Unité de Biologie des Bactéries pathogènes à Gram-positif, Institut Pasteur, Paris, France; ^2^UMR 3525*,* CNRS, Paris, France; ^3^Université Pierre et Marie Curie, Paris, France; ^4^Laboratoire de Microbiologie et Génétique Moléculaires, CNRS, Toulouse, France

**Keywords:** transposase, integrative and conjugative element, insertion sequence, evolution, genome dynamics

## Abstract

Transposable elements (TEs) are major components of both prokaryotic and eukaryotic genomes and play a significant role in their evolution. In this study, we have identified new prokaryotic DDE transposase families related to the eukaryotic Mutator-like transposases. These genes were retrieved by cascade PSI-Blast using as initial query the transposase of the streptococcal integrative and conjugative element (ICE) Tn*GBS2*. By combining secondary structure predictions and protein sequence alignments, we predicted the DDE catalytic triad and the DNA-binding domain recognizing the terminal inverted repeats. Furthermore, we systematically characterized the organization and the insertion specificity of the TEs relying on these prokaryotic Mutator-like transposases (p-MULT) for their mobility. Strikingly, two distant TE families target their integration upstream σ_A_ dependent promoters. This allowed us to identify a transposase sequence signature associated with this unique insertion specificity and to show that the dissymmetry between the two inverted repeats is responsible for the orientation of the insertion. Surprisingly, while DDE transposases are generally associated with small and simple transposons such as insertion sequences (ISs), p-MULT encoding TEs show an unprecedented diversity with several families of IS, transposons, and ICEs ranging in size from 1.1 to 52 kb.

## Introduction

Since their discovery by Barbara McClintock in the 1940s, transposable elements (TEs) have gradually attracted increasing interest. TEs were first thought to be potentially harmful parasitic entities and now are recognized as major contributors to genome evolution. TEs have been found in nearly all sequenced organisms where they can represent an important proportion of the host genome. A recent analysis of the annotation of 10 million protein-encoding genes in sequenced eukaryotic, archaeal, bacterial, and viral genomes and metagenomes revealed that transposases are the most abundant and the most ubiquitous genes in nature (Aziz et al. 2010).

Transposases, the enzymes catalyzing transposition of DNA segments, are classified in phylogenetically and structurally unrelated families, and DDE transposases represent one of the major classes. DDE transposases show similar catalytic domain architectures with a conserved triad of essential amino acids (Asp, Asp, and Glu) which coordinates a divalent metal ion (Haren et al. 1999; Curcio and Derbyshire 2003). This catalytic domain is associated with a DNA-binding region generally located in the N-terminal part of the protein responsible for the recognition of the terminal inverted repeats (IRs) at the TE extremities which correctly localizes the transposase for strand cleavage and transfer in the transposition reaction. DDE transposases are present in all three domains of life: eukaryotes, eubacteria, and archaea. Indeed, the integrases of retroviruses and that of the *Escherichia coli* bacteriophage mu represent extensively studied classes of DDE transposases (Montano et al. 2012). Although DDE transposases are structurally linked, they show an overall low sequence conservation leading to numerous unannotated or misannotated representatives in genome databases. In eukaryotes, comparative analysis of DDE transposases led to the identification of 19 superfamilies (Jurka et al. 2005). A recent phylogenetic analysis suggested that all eukaryotic cut-and-paste transposable element superfamilies have a common evolutionary origin and define three major phyla (Yuan and Wessler 2011). Among these groups, the highly mutagenic Mutator and Mutator-like elements (MULEs) represent a diverse family that is related to the prokaryotic IS*256* family (Eisen et al. 1994).

In prokaryotes, insertion sequences (ISs) are the simplest and the most abundant autonomous TEs. They were defined as TEs that only code the functions required for their mobility: a transposase gene surrounded by IRs that define the borders of the mobile DNA. IS have a dedicated repository database, ISfinder (www-is.biotoul.fr, last accessed January 27, 2014), that contains more than 4,000 carefully annotated ISs (Siguier et al. 2006). Some prokaryotic TEs harbor different “passenger genes,” implicated in regulatory or accessory functions such as antibiotic resistance genes which confer a selective advantage to the host. However, the organization of these transposons may be even more complex. We have recently characterized a new family of TEs in streptococci, the Tn*GBS* family, encoding a DDE transposase associated with different conjugative machineries that promote their horizontal transfer (Brochet et al. 2009; Guerillot et al. 2013). This represents the first family of integrative and conjugative elements (ICEs) in which the phage-like integrase responsible for the excision and integration of the element is substituted by a DDE transposase. Tn*GBS*s were shown to transpose specifically 15–17 bp upstream different σ_A_ promoters (Brochet et al. 2009). Similarity searches of the public databases revealed IS elements expressing transposases related to Tn*GBS* transposases (Brochet et al. 2009). These enzymes were not related to any known transposase.

Here, by a cascade iterated Blast search we have expanded our vision of the diversity of p-MULT with the discovery of four new families, in addition to the IS*256* family. Tn*GBS* and related ISs transposases represent one of these new families. Similarity and secondary structure predictions allowed us to determine that these five families share an RNase H fold and to identify, unambiguously, the catalytic triad as well as the IR DNA-binding regions. By systematic analysis of the genomic context we showed that these transposases are responsible for the mobility of ISs both in eubacteria and in archaea but also of ICEs previously described in *Mycoplasma* (Dordet Frisoni et al. 2013). The identification of a new family of Mutator-like elements sharing the same insertion specificity as Tn*GBS* upstream σ_A_ promoters provides further insights into this unusual property among prokaryotic transposases.

## Materials and Methods

### Cascade PSI-Blast Search of Transposases Related to Gbs1118

The primary protein sequence of Tn*GBS2* transposase (Gbs1118) was used as an initial query in a PSI-Blast (Altschul et al. 1997) search against the NCBI nonredundant protein sequence database. Two rounds of PSI-Blast searches were performed without low complexity filter and with otherwise default parameters. Protein hits with an *E*-value above 0.005 and query coverage <60% were filtered out. Retained hits were then aligned using the MAFFT algorithm (Katoh and Standley 2013) with default parameters and a tree was built with Jalview using the average distances calculated with the BLOSUM62 matrix (Waterhouse et al. 2009). Based on this tree, a protein hit distantly related to the query was chosen to perform a second PSI-Blast search. New protein hits obtained by this second round of PSI-Blast were retained and a new query for subsequent rounds of PSI-Blast search was selected by applying the same filtering, alignment, and tree building method. The systematic propagation of PSI-Blast searches through distantly related homologs allowed us to overcome the query dependence and asymmetry of the classical use of PSI-Blast (Bhadra et al. 2006). In total, we performed seven rounds of PSI-Blast search with the following queries: Gbs1118 (NP_735564), Hore_07130 (YP_002508465), Krac_8686 (ZP_06969815), MAE_08640 (YP_001655878), MAGa5060 (YP_003515670), NAS141_01721 (ZP_00964747), and Calow_0284 (YP_004001685).

### Transposase Family Clustering

Protein hits retrieved by cascade PSI-Blast were first compared by all-against-all BlastP comparisons. Similarities with an *E*-value lower than 10^−^^4^ were retained to build a similarity network using the Cytoscape software (Cline et al. 2007). We applied the force directed layout of Cytoscape to visualize the generated similarity graph where each node corresponds to a transposase homolog interconnected by edges representing the BlastP results. We applied a continuous mapping of the edge opacity to further weigh relationships between transposase homologs (the more opaque edges correspond to lower BlastP *E*-values). Transposase homologs were then clustered by using the Markov cluster algorithm (MCL) (http://micans.org/mcl/, last accessed January 27, 2014) implemented in the clusterMaker plugin of Cytoscape (Morris et al. 2011). We converted the edge weight with −log(*E*-value) and applied an inflation factor (IF) of 1.2. This inflation value was chosen as it has been shown to be effective in clustering other well-defined IS families (Siguier et al. 2009).

### Identification of Transposable Elements and of Their Insertion Sites

The identification of TEs was performed semiautomatically by using scripts written in Python programming language and using the Biopython module (www.biopython.org/, last accessed January 27, 2014). First, the DNA coding sequences of all transposase homologs were retrieved together with 400 bp of up- and downstream sequences. The extracted DNA sequences were then used as BlastN queries against the complete or draft genome sequences in which the TE is inserted. If the BlastN result gave more than three hits, the most likely TE boundaries were determined automatically based on the majority start and end of high-scoring segment pairs (hsp). BlastN results giving multiple hsp that align with the first base of the query likely correspond to larger TEs encoding several open reading frames (ORFs) upstream the transposase gene. For these transposases, the length of the surrounding DNA sequence was extended until the extremities of the TE were reached. For transposases present in less than three copies, the extracted DNA sequence was compared by BlastN with the genomic sequence of other isolates from the same species, if available. All TE boundaries were manually validated by the identification of IRs and direct repeats (DRs). All transposons and ISs identified in this study (supplementary table S1, Supplementary Material online) were submitted to the ISfinder database (Siguier et al. 2006).

Insertion sites and insertion specificity were analyzed upon extracting 300 bp sequences on both sides of the validated TEs after filtering identical insertions. These regions were scanned for putative σ_A_ promoters using the PPP software (http://bioinformatics.biol.rug.nl/websoftware/ppp, last accessed January 27, 2014). Hidden Markov models of the lactococcal σ_A_ dependent RNA polymerase binding site, allowing a 15–19 bp distance between the canonical −35 and −10 promoter elements, were constructed using alignments of known σ_A_ binding sites (Zomer et al. 2007).

### Phylogeny and Transposase Sequences Analysis

For phylogenetic reconstruction, transposase sequences with BlastP similarity lower than 98% and representative of the diversity of TEs were retained. The transposase sequences were aligned using MAFFT version 6 with the E-INS-I method (Katoh and Standley 2013) and manually checked using Jalview (Waterhouse et al. 2009). Aligned positions with more than 60% of gaps were removed before constructing the tree. Phylogenetic relationships were inferred by Maximum likelihood (ML) using MEGA5 (Tamura et al. 2011). Prior to ML analysis, the best protein substitution model of Jones-Taylor-Thornton (JTT) was selected according to the Akaike information criterion given by the ProtTest software (Darriba et al. 2011). Branch support was determined by 100 bootstrap replications. The level of conservation in protein sequence alignments was plotted using the plotcon application (http://emboss.sourceforge.net/, last accessed January 27, 2014). Secondary structure predictions were performed using the jpred3 server http://www.compbio.dundee.ac.uk/jpred (last accessed January 27, 2014) (Cole et al. 2008).

## Results

### Discovery of New Families of Mutator-Related Transposases

Transposases from Tn*GBS*s and related ISs show a PFAM *rve* retroviral integrase domain with a low score (Brochet et al. 2008). However, we did not retrieve known transposase sequences by BlastP search at NCBI or at the ISfinder Web site. BlastP shows a low sensitivity. To retrieve more distantly related protein sequences, we performed a cascade PSI-Blast search in the nonredundant protein database. After applying the filters described in the Materials and Methods section, we retained 731 protein hits. Interestingly, although 70% of these (517) are currently described as hypothetical proteins, 104 were annotated as Mutator-like transposases, 23 as transposases of the IS*256* family, 7 as IS*H6* transposases, and 80 as transposases of unknown families. This iterative search suggests that, contrary to our first analysis, Tn*GBS* transposases are distantly related to known transposase families.

**Fig. 1.—:**
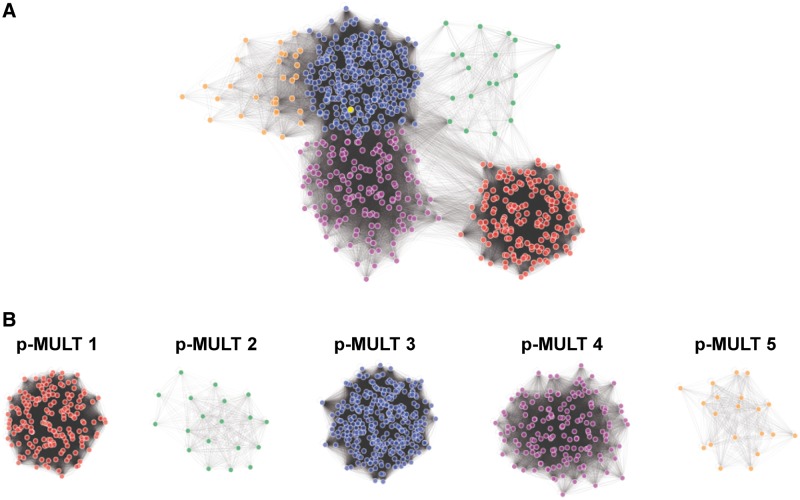
Similarity network and MCL clustering of prokaryotic Mutator-like transposases. (*A*) Similarity network of Tn*GBS* transposases related protein sequences weighted according to all-against-all BlastP *E*-values. Each node represents a protein sequence obtained by the cascade PSI-Blast search. More opaque edges correspond to greater similarity according to the BlastP *E*-value. Each node was colored according to the MCL clustering of the network. (*B*) Similarity network of the five p-MULT families defined by MCL clustering of the all-against-all similarity graph of figure 1*A*.

**Fig. 2.—:**
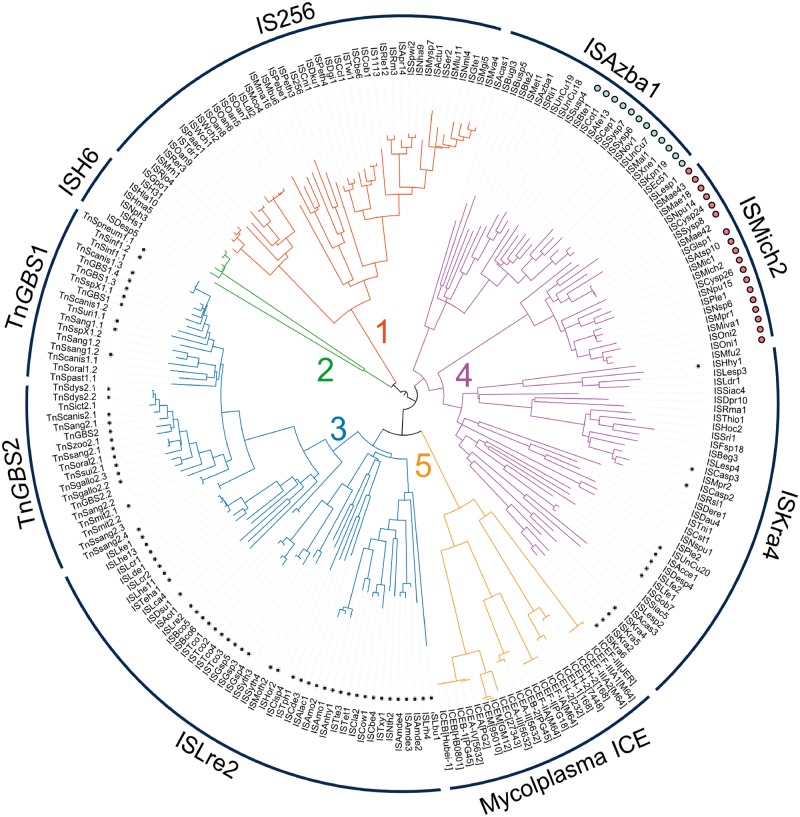
Phylogenetic tree of prokaryotic Mutator-like transposases. Each p-MULT clade is colored according to figure 1. p-MULT 1 and p-MULT 2 transposases are encoded by ISs of the IS*256* and IS*H6* families, respectively. p-MULT 3 transposases are encoded by both the Tn*GBS* family and the IS*Lre2* family. p-MULT 4 encoded by both transposons and by ISs form three different lineages: IS*Azba1*, IS*Mich2*, and IS*Kra4*. Transposons of the IS*Azba1* group encoding a pRiA4_Orf3-like protein are indicated by blue dots. IS of the IS*Mich2* group with a predicted −1 frameshift in the transposase gene are indicated by pink dots. TE names are indicated at the extremity of the tree branches. TEs with a predicted σ_A_ promoter at a distance of 13–17 bp from the IR-genome junctions in more than 20% of their insertion sites (supplementary table S2, Supplementary Material online) are indicated by small black dots.

We then built a similarity graph to visualize the relatedness between protein sequences and to decipher the overall relationships of putative and characterized transposases. In this network, protein sequences are represented as nodes that are connected by edges weighted according to their BlastP *E*-Value (fig. 1*A*). All hits form an interlinked network in agreement with the overall relatedness of all protein hits observed in the course of the PSI-Blast analysis. In particular, it shows the relatedness of Tn*GBS* transposases with the Mutator transposase superfamily and transposase of the previously identified IS*256* and IS*H6* families. By using the MCL, the protein sequences of the similarity network were clustered in five groups, each defining a family of transposases that we named p-MULT 1–5 for prokaryotic Mutator-like transposase. Proteins previously identified as transposases of the IS*256* and IS*H6* families are members of two different clusters, p-MULT 1 and p-MULT 2. Tn*GBS* transposases are members of a large cluster encompassing 320 proteins (p-MULT 3) that are closely linked to two others clusters of 186 (p-MULT 4) and 31 (p-MULT 5) proteins, respectively (fig. 1). Strickingly, except for the putative p-MULT 5 transposases that are only connected to Tn*GBS* transposases (p-MULT 3), the four other clusters are interconnected. The phylogenetic tree constructed with representatives of each family confirms this clustering (fig. 2). Our analysis extends a previous study reporting that IS*H6* transposases are distantly related to the IS*256* family (Filee et al. 2007). The discovery of three additional putative transposase families shows that, together with IS*256* and IS*H6* transposases, Mutator-like transposases in prokaryotes are much more diverse and widespread than previously thought.

### p-MULTs Are Encoded by Diverse Types of Mobile Elements: ISs, Transposons, and ICEs That Share Transposition Features

**Fig. 3.—:**
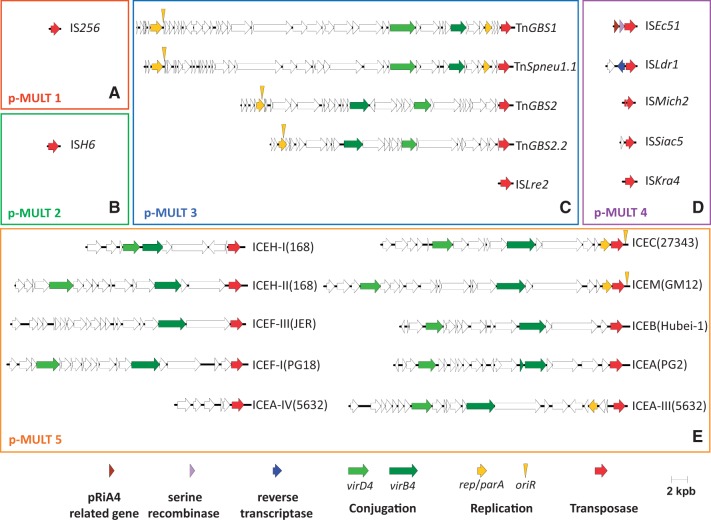
Diversity of transposable elements encoding transposases of the five p-MULT families. Representation of the gene maps and structural/organizational diversity of the five TEs families: (*A*) p-MULT 1, IS*256* family; (*B*) p-MULT 2, IS*H6* family; (*C*) p-MULT 3, Tn*GBS*/IS*Lre2* family; (*D*) p-MULT 4, IS*Azba1*, IS*Mich2*, and IS*Kra4* families; (*E*) p-MULT 5 Mycoplasma ICE family. Arrows represent genes. Predicted functions of the gene products are indicated according to a color code shown at the bottom of the figure. Putative origins of replication are represented by yellow triangles.

To systematically identify the TEs that encode transposases of the five p-MULT families, we analyzed the DNA regions on both sides of the transposase genes for IRs and direct repeats (DRs). In total, we accurately identified 424 TEs in addition to the 58 Tn*GBS* related ICE previously described (supplementary table S1, Supplementary Material online). The 109 TEs encoding a p-MULT 1 transposase have the genetic organization of IS (fig. 3*A*). Based on BLAST analysis performed on the ISfinder database, they all belong to the IS*256* family. These ISs are widely distributed among bacterial phyla (Proteobacteria, Firmicutes, Chlamydiae, Actinobacteria, and Deferribactere) and are also present in the archaeal phylum Euryarchaeota. Similarly, the 11 TEs that encode IS*H6* related transposases (p-MULT 2) are ISs (fig. 3*B*). Six are new representatives of this small group. Interestingly, although the IS*H6* group was first identified in archaea of the Euryarchaeota phylum (Filee et al. 2007), we identified multiple copies of one IS belonging to this family in three different uncultured *Desulfobacterium* strains that are members of the proteobacterial phylum (IS*Desp5*, fig. 2).

In the three other families, we identified more complex TEs. TEs encoding p-MULT 3 transposases include the Tn*GBS* ICEs and 168 ISs that form a new IS family that we named IS*Lre2* (fig. 3*C*). Unlike Tn*GBS* that are restricted to the streptococcal genus, IS*Lre2* ISs were found in a broad variety of Firmicute species, and eight are present in multiple copies in a *Fusobacterium* and two *Synergistetes* strains, respectively, belonging to two distantly related phyla.

The 115 TEs encoding p-MULT 4 transposases were found in numerous phyla: Proteobacteria, Cyanobacteria, Nitrospirae, Bacteroidetes, Actinobacteria, Planctomycetes, and Chloroflexi. According to the transposase phylogeny, three distinct groups of ISs or simple transposons, that we named IS*Azba1*, IS*Mich2*, and IS*Kra4*, are clearly distinguishable. Among the IS*Azba1* group, 12 TEs forming a monophyletic branch harbor one to three ORFs in addition to the transposase gene (figs. 2 and 3*D*). They share an ORF similar to one of unknown function in *Agrobacterium rhizogenes* plasmid pRiA4 (Endoh et al. 1990). Among these TEs, five carry a serine recombinase gene and one a tyrosine recombinase gene, respectively (supplementary table S1, Supplementary Material online). As shown for the Tn*3* family, these site-specific recombinases might be involved in the resolution of cointegrates generated by the transposition (Kostriken et al. 1981). Similarly, four IS*Kra4* elements encode different genes of unknown function in addition to their transposases (fig. 3*D* and supplementary table S1, Supplementary Material online). IS*Ldr1* from *Legionella drancourtii* LLAP12 encodes a group II intron putative reverse transcriptase (fig. 3*D*). Conversely, all IS*Mich2* TEs are IS. However, in this group, the transposases are encoded by two ORFs, and a −1 frameshift is sufficient to restore the expression of a full-length transposase. As this feature is conserved in the IS*Mich2* group, it likely reflects a mode of regulation of transposition by programmed translational frameshifting as described in the IS*1* or IS*3* families (Chandler and Fayet 1993; Nagy and Chandler 2004).

The 31 TEs encoding p-MULT 5 transposases were identified in the genomes of *Mycoplasma* species. We precisely characterized the boundaries of 21 elements. Surprisingly, they are all of large size, ranging from 7 to 37 kb (fig. 3*E*). Eight of these are present in two or three copies. Except for the 7-kb-long element, they all encode homologs of type IV secretion systems proteins responsible for the mobility of conjugative elements (fig. 3*E*). Thirteen of these elements were previously predicted as ICE despite the absence of an identifiable integrase gene. The protein responsible for their integration was not known (Marenda et al. 2006). These ICEs share little sequence conservation and have diverse organizations, as illustrated by ten representatives depicted in figure 3*E*. Strikingly, the most unifying feature of these elements is the conservation of a p-MULT 5 putative transposase gene upstream of one of the two IR. This strongly suggests a role for this transposase in *Mycoplasma* ICE mobility. This prediction was recently experimentally demonstrated for ICEA of *Mycoplasma agalactiae* 5632 (Dordet Frisoni et al. 2013). ICEA is transferable by conjugation and its excision and integration involve a p-MULT 5 transposase encoded by CDS22. These ICEs, like the Tn*GBS*s, therefore rely on a DDE transposase of the Mutator family and not a tyrosine or serine recombinase for their mobility. Based on the identification of the putative transposase gene, we identified five new ICEs in three *Mycoplasma hyopneumoniae* strains and one in *Mycoplasma capricolum* subsp. *capricolum* strain ATCC 27343.

Despite their genetic diversity, TEs encoding p-MULT transposases share several features. We identified 18- to 39-bp-long IRs at their extremities with a conserved terminal cytosine residue (supplementary fig. S1*A*, Supplementary Material online). Like eukaryotic MULEs, the insertion of these prokaryotic TEs generates DRs of 8 or 9 bp (supplementary table S1, Supplementary Material online). We have shown that Tn*GBS*s transpose by production of an extrachromosomal circular form, which acts as a substrate for a plasmid-like replication and conjugative transfer. Similar circular forms of IS*Lre*2 in *Lactobacillus reuteri* JCM 1112 have been detected by polymerase chain reaction (data not shown). IS*256* family ISs also transpose via a circular intermediate (Loessner et al. 2002) as do *Mycoplasma* ICEA and ICEF-I. In these circular forms, the terminal IRs are separated by 6–10 bp sequences derived from one of its two flanking DNA sequences (Calcutt et al. 2002; Marenda et al. 2006; Brochet et al. 2009; Dordet Frisoni et al. 2013; Guerillot et al. 2013). These data suggest that the Mutator-like transposases of the five families catalyze transposition using a similar mechanism involving the formation of a circular intermediate.

### Insertion Specificity for Upstream Promoter Regions Is Shared by the p-MULT 3 Family and One Lineage of p-MULT 4

**Fig. 4.—:**
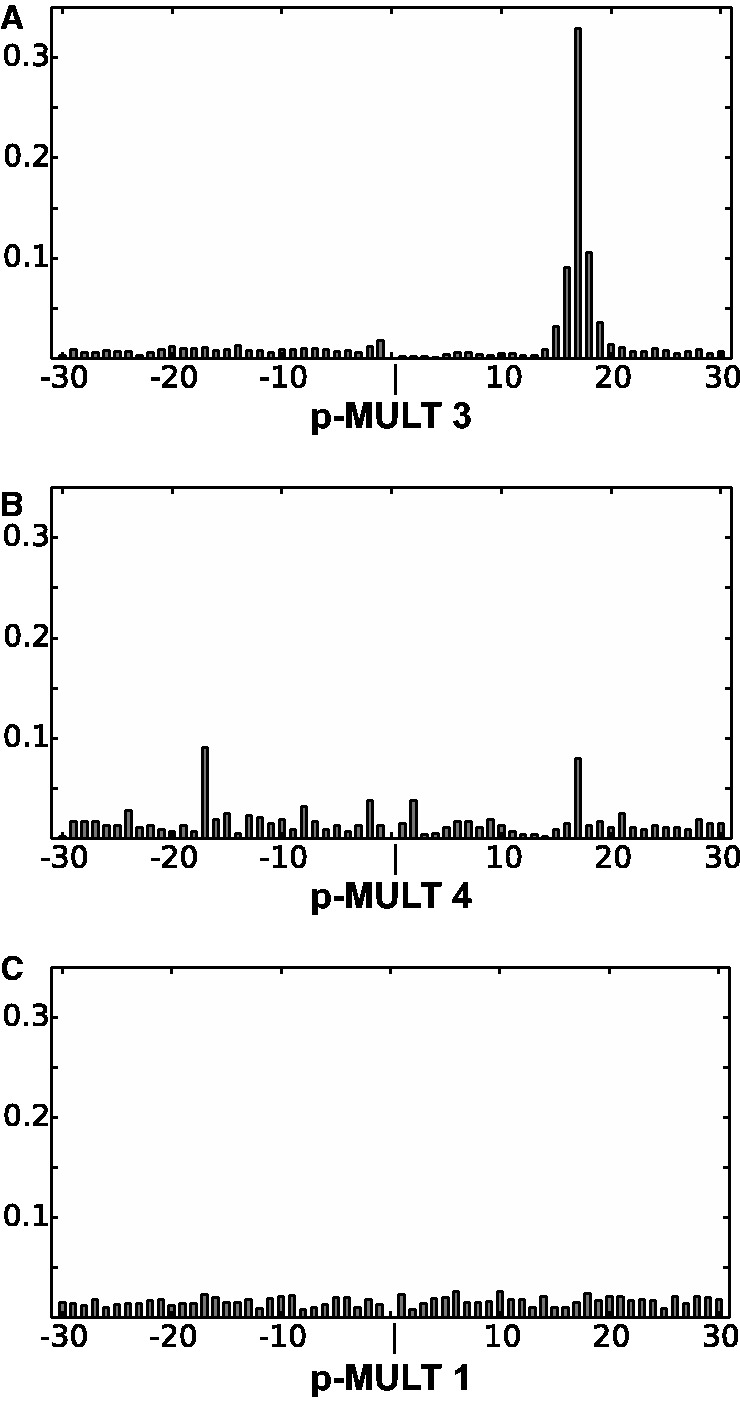
Relative position of putative σ_A_ promoters identified in the DNA region surrounding TE insertion sites. The DNA sequences on both sides of insertions were extracted and scanned for putative σ_A_ promoters using the PPP software (Zomer et al. 2007). The histogram represents the ratio of the predicted σ_A_ promoters at a given position from the insertion site to the total number of σ_A_ promoters predicted at a maximum distance of 30 bp. Only the results obtained for p-MULT 3 (*A*), p-MULT 4 (*B*), and p-MULT 1 (*C*) for which a sufficient number of promoters were predicted are represented (4,207, 523, and 1,145 σ_A_ promoters, respectively). The abscise numbers correspond to the position of the predicted −35 sequence of σ_A_ promoters relative to the insertion site. Negative values correspond to the IRl–genome junctions and positive values to IRr–genome junctions.

We performed a systematic analysis of the insertion specificity of the five p-MULT families by extracting cognate genomic DNA sequences next to the IR-right and left of each TE (supplementary table S2, Supplementary Material online). In total, we obtained 2,833 IR-genomic DNA junctions. Tn*GBS* and the related ISs are preferentially inserted 15–17 bases upstream the −35 region of σ_A_ promoters (Brochet et al. 2009; Guerillot et al. 2013). To determine whether other p-MULT families show a similar insertion specificity, we first searched for putative σ_A_ promoter sequences on both sides of the TEs (supplementary table S2, Supplementary Material online). We then analyzed the position-specific enrichment of promoter detection relative to the end of the IRs. The result of this analysis is depicted in figure 4 for the insertion sites of TEs encoding p-MULT 1, 3, and 4. For the p-MULT 3 family, a strong relative increase of promoter detection is observed at a distance of 16 bp from IR-right (fig. 4*A*), whereas no enrichment at any specific position was detected for TEs encoding p-MULT 1 transposases (fig. 4*C*). This confirms the oriented insertion at a fixed distance from σ_A_ promoters catalyzed by p-MULT 3. More interestingly, we detected a similar but lower signal for TEs encoding p-MULT 4 transposases (fig. 4*B*). Therefore, some p-MULT 4 transposases share the p-MULT 3 insertion specificity for upstream promoter regions (supplementary tables S1 and S2, Supplementary Material online). However, σ_A_ promoters were predicted on both sides of the element. These 13 ISs (IS*Kra2*, *4*, *5* and *6*; IS*Casp2* and *3*, IS*Lfe1* and *2*; IS*Hhy1*; IS*Acce1*; IS*Tvi*1; IS*Uncu*20; and IS*Desp*4) are characterized by an almost perfect complementarity (83–100%) between the right and left IRs. Likewise, IS*Lbu1* encoding a p-MULT 3 transposase of *Leptotrichia buccalis* shows perfectly complementary IR of 24 bp and was the only TE of this family found inserted in both orientations with respect to σ_A_ promoters (supplementary table S2, Supplementary Material online). These observations suggest that the orientation of TE insertions relies on a differential recognition of the two IRs by the transposase during integration.

We did not observe any conserved DNA motif in the DNA flanking the insertions of TEs encoding p-MULT 1 or p-MULT 5 transposases, in agreement with previous observations showing that insertion of IS*256* and *M. agalactiae* ICEA is likely random (Ziebuhr et al. 1999; Dordet Frisoni et al. 2013). For the TEs encoding p-MULT 2 transposases, we observed a conservation of the flanking region among the 39 IS-genome junctions extracted. The consensus sequence of the DR corresponds to the AT-rich motif AANATNTT (supplementary fig. S1*B*, Supplementary Material online). Remarkably, we observed this sequence also at the insertion sites of the distantly related IS identified in the uncultured *Desulfobacterium* sp. (IS*Desp5*). The conservation of this particular targeting further supports the grouping of these bacterial ISs as new members of the IS*H6* family.

### Conservation of a Mutator-Like Catalytic Domain

**Fig. 5.—:**
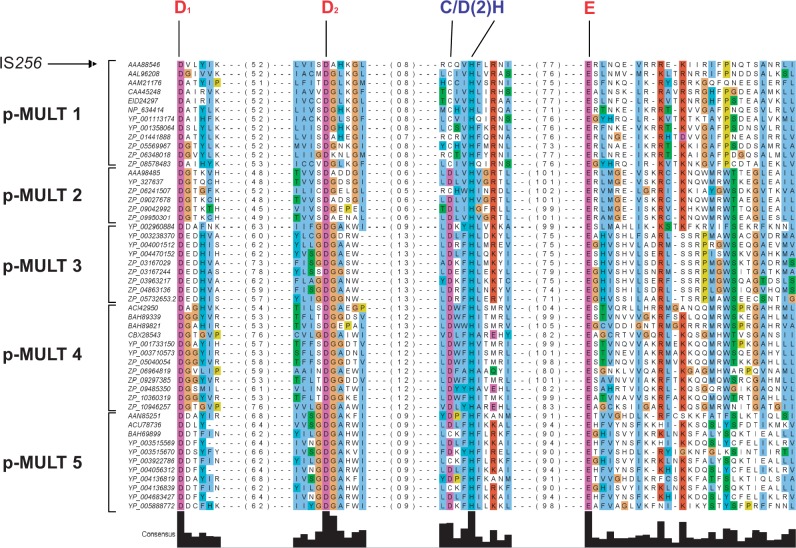
Alignment of the protein domains encompassing the catalytic DDE residues in p-MULT. Transposase sequences were aligned by the MAFFT alignment software (Katoh and Standley 2013) and visualized using Jalview (Waterhouse et al. 2009). The alignment was filtered for redundancy to subsequently retain a subset of transposases for each p-MULT family representative of their diversity. Only regions surrounding the predicted DDE residues and the C/D(2)H motif were kept in the alignment. Numbers given in parentheses correspond to the distance in aa residues between the different motifs. Transposases accession numbers are indicated on the left.

The catalytic triad of DDE transposases consists of two aspartyl (D) residues and a glutamyl (E) residue, located in a conserved core that forms a characteristic RNase H-like fold of mixed α-helices and β-strands (see supplementary fig. S2*A*, Supplementary Material online) (Hickman et al. 2010). The first D residue is located in β1, the second D residue is in or just after β4, and the third D/E residue in or just before α4. These three catalytic residues were experimentally confirmed in the IS*256* transposase (Loessner et al. 2002). To identify the catalytic residues in the five p-MULT families, we combined sequence alignments and secondary structure predictions. First, we observed that the sequence of the transposases from the five p-MULT families align perfectly at the three catalytic residues of the IS*256* transposase (fig. 5). Second, secondary structure modeling of representatives of the five transposase families unraveled an RNase H-like fold with the expected positioning of the conserved DDE residues (supplementary fig. S2*A*, Supplementary Material online), despite divergences in the regions between these putative catalytic residues. Compared with a typical RNase H fold, some DDE transposase catalytic domains are characterized by the presence of a β-strand or a α-helical insert between the second D residue and the E residue (Hickman et al. 2010; Yuan and Wessler 2011). For the five p-MULT families, a 99- to 138-aa-long α-helical insert was predicted between the catalytic residues D2 and E, like previously shown in eukaryotic Mutator transposases (Hua-Van and Capy 2008; Yuan and Wessler 2011). Altogether these results allowed us to predict with a high confidence the three catalytic residues in the transposases of the five p-MULT families (fig. 5).

In addition to the predicted catalytic DDE residues, a specific signature (C/D(2)H) is conserved in all retrieved homologs, 11–19 aa downstream D2 (fig. 5). This motif is positioned in the α-helical insert located after the predicted strand-β5 in the five p-MULT families (supplementary fig. S2*A*, Supplementary Material online). Although the functional role of this motif is unknown, it has also been identified with a similar relative position in the predicted α-helical insert of eukaryotic Mutator-like transposases (Yuan and Wessler 2011).

### Prediction of an N-Terminal DNA-Binding Domain Implicated in IR Recognition

**Fig. 6.—:**
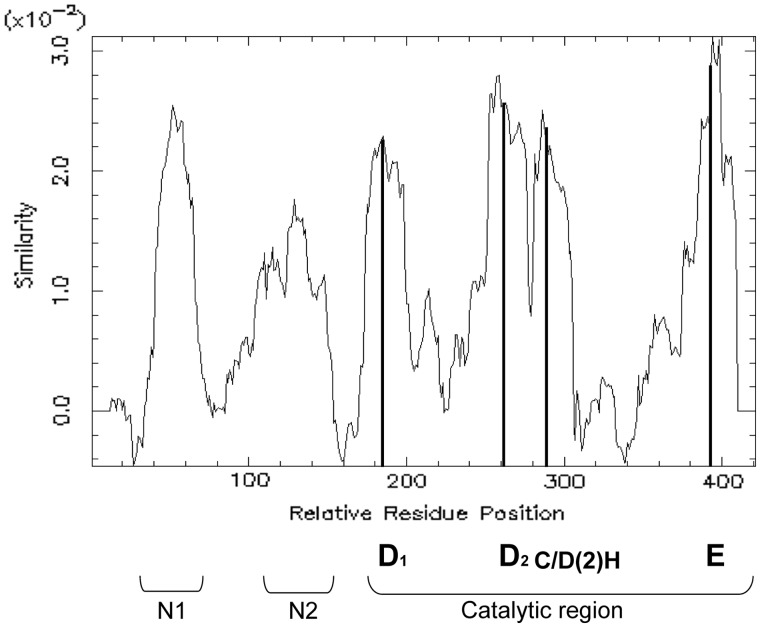
Level of conservation along the alignment of p-MULT sequences. The graphical representation of the similarity scores along the aligned p-MULT sequences was plotted using the plotcon software of the EMBOSS package (www.ebi.ac.uk/Tools/emboss/, last accessed January 29, 2014). The similarity was calculated by moving a window of 15 aa residues along the aligned sequences. The similarity score at each position corresponds to the average of all the possible pairwise scores at that position. The pairwise scores are taken from the BLOSUM62 matrix. The average of the similarity values at each position within the window was plotted.

**Fig. 7.—:**
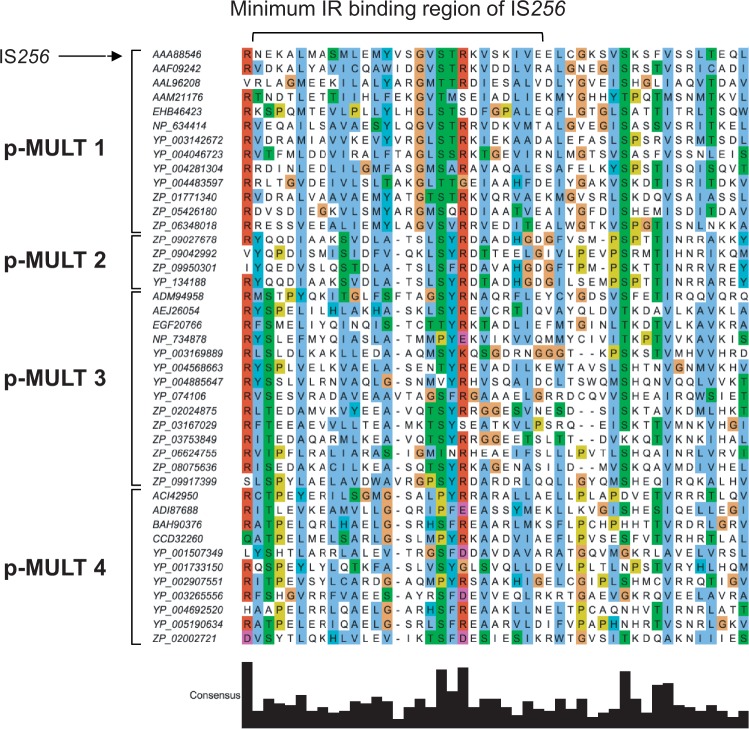
Alignment of the predicted N-terminal DNA binding domain implicated in p-MULT IR recognition. Transposases identified in this study were aligned by the MAFFT alignment software (Katoh and Standley 2013) and visualized using Jalview (Waterhouse, et al. 2009). Only the predicted N2 domain encompassing the minimum IR-binding domain identified in the IS*256* transposase (Hennig and Ziebuhr 2010) was retained in the alignment. The alignment was filtered for redundancy in order to keep a subset of transposases representative of the diversity of p-MULT 1, 2, 3, and 4 families. Transposases accession numbers are indicated on the left.

Two additional domains are conserved in the N-terminal part of the alignment of the p-MULT sequences (domain N1 and N2 in fig. 6 showing the similarity along the aligned sequences). Domain N2, situated upstream the catalytic domain, corresponds to the DNA-binding region shown to recognize the terminal IRs of IS*256* (fig. 7) (Hennig and Ziebuhr 2010). For the five transposase families, an identical secondary structure of two α helices of similar length separated by a possible turn formed by three residues were predicted in this region, and several conserved residues are identically positioned in this structure (supplementary fig. S2*B*, Supplementary Material online). Therefore, this region probably represents the IR binding region for the five families.

However, in 5 of the 31 putative transposases of *Mycoplasma* ICE, we also found a more divergent domain at the same position matching the HTH_23 PFAM domain (supplementary table S1, Supplementary Material online). This domain is present in transcription regulators but has also been identified in the DDE transposase of the IS*630* (equivalent to the mariner family) and IS*30* families (Nagy et al. 2004). This observation suggests a replacement of the N2 domain in these five transposases.

### Insertion Specificity Upstream Promoters Is Associated with a Specific Transposase Sequence Signature

**Fig. 8.—:**
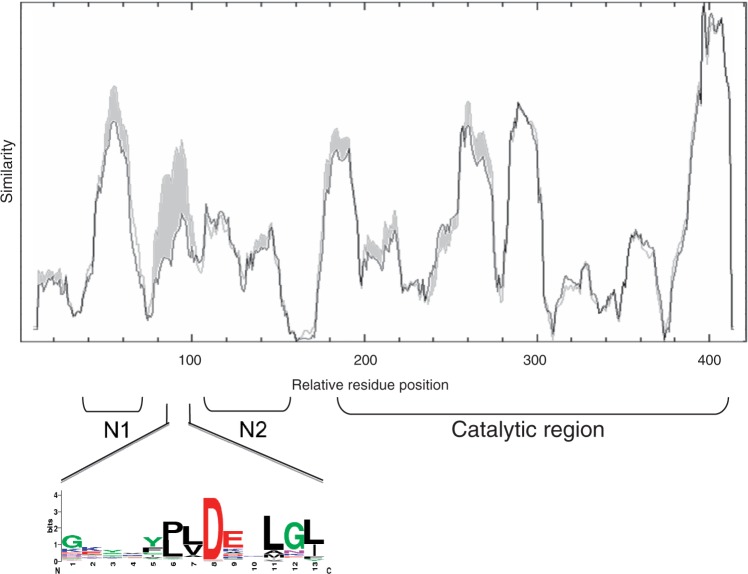
Transposase sequence signature associated with insertion specificity upstream σ_A_ promoters. Like in figure 6, the similarity score along the aligned putative transposase sequences was plotted from the MAFFT alignment using the plotcon software of the EMBOSS package. The similarity was calculated by moving a window of 10 aa residues along the aligned sequences and using the BLOSUM62 matrix. Two similarity plots were superimposed. The gray and black curves correspond to the similarity calculated on the alignment of p-MULT 3 plus IS*Kra4* transposases (p-MULT 4) and of p-MULT 3 plus IS*Azba1* and IS*Mich2* transposases (p-MULT 4), respectively. The regions showing a higher conservation with IS*Kra4* transposases than with IS*Azba1* and IS*Mich2* transposases are colored in gray. The protein motif of the region specifically conserved between p-MULT 3 and p-MULT 4 transposases of the IS*Kra4* group, and putatively involved in the targeting of σ_A_ promoters, was generated by using WebLogo (Crooks et al. 2004).

p-MULT 4 transposases belong to three different phylogenetic lineages. We took advantage of the fact that all p-MULT 4 transposases that catalyze integration of their cognate element upstream of putative σ_A_ promoters belong to the IS*Kra4* group (fig. 2) to search for particular motifs associated with this insertion specificity. We compared the similarity of p-MULT 3 with p-MULT 4 transposases of the IS*Kra4* group or of the IS*Azba1* and IS*Mich2* groups (fig. 8) and identified a single region located between the conserved domains N1 and N2 which is differentially conserved. This region contains a conserved aspartyl residue (fig. 8). Comparison with transposases from the other p-MULT families showed that this motif is conserved only between the Tn*GBS*, IS*Lre2*, and IS*Kra4* transposases that catalyze insertion upstream σ_A_ promoters. This strongly suggests that the motif is involved in insertion specificity.

## Discussion

Mutator and Mutator-like transposases are one of the major superfamilies of transposases in eukaryotes (Yuan and Wessler 2011). They are encoded by diverse TEs present in most eukaryotic lineages, including mammals, plant, fungi, and amoeba (Hua-Van and Capy 2008). The name Mutator originates from the ability of active copies of this element to induce mutations corresponding to diverse recombination events, which were first described in plants (Bennetzen 1984; Jiang et al. 2011) and later in other organisms (Amyotte et al. 2012). Among bacterial TEs, the IS*256* family was shown to be related to the eukaryotic Mutator-like elements (Mahillon and Chandler 1998; Hua-Van and Capy 2008). In this study, we further expanded the Mutator transposase superfamilies in prokaryotes by the discovery of four additional families related to IS*256*, defining five p-MULT families (fig. 1). The majority of prokaryotic DDE transposases are associated with IS. Although the IS*256* (p-MULT 1) and IS*H6* (p-MULT 2) families contain only ISs, the three other Mutator-like families display a wide variety of different organizations (fig. 3). TEs encoding p-MULT 3 transposases include ISs and the diverse streptococcal ICEs of the Tn*GBS* family (Guerillot et al. 2013). More interestingly, all TEs encoding p-MULT 5 transposases are *Mycoplasma* ICEs previously described or identified in this study (Marenda et al. 2006).

The only other former example of an association between a DDE transposase and a conjugation machinery is ICE*6013* (Han et al. 2009; Smyth and Robinson 2009). The combination of transposition with conjugation implies recombination constraints linked to the physical separation of donor and recipient molecules. The expansion of ICE families relying on different IS related DDE transposases highlights that transposition via a circular intermediate overcomes these constraints by generating a molecular substrate compatible with the conjugative transfer. As this mode of transposition is common to several widespread families of IS, such as IS*1*, IS*3*, IS*21*, and IS*30* (Polard et al. 1992; Turlan and Chandler 1995; Kallastu et al. 1998; Kiss and Olasz 1999; Berger and Haas 2001), the association of transposition with conjugative transfers of DNA might be underestimated. Alternatively, the transposition process catalyzed by Mutator-like transposases might be particularly adapted to conjugative transfer explaining why they are associated with two broad families of ICEs.

Tn*GBS*s were shown to replicate both in the donor strain following circularization and in the recipient strain upon their insertion in the chromosome. This replication is dependent on a plasmid-like replicase and promotes the transfer of the ICE (Guerillot et al. 2013). *Mycoplasma* ICEs show several features suggesting a transient replication. First, in ICEC_(27343)_, ICEM_(95010)_, and ICEM_(GM12)_, a sequence of 70–286 nt located between the putative tranposase gene and the terminal IR shows more than 83% identity with the DNA region containing the putative single strand origin of replication of the plasmid pMmc-95010 (Thiaucourt et al. 2011). Second, we identified in ICEC_(27343)_, ICEM_(GM12)_, and ICEA-III_(5632)_ a *parA* homolog (fig. 3*E*). ParA proteins are implicated in the segregation of replicating plasmids (Lutkenhaus 2012). Therefore, a transient replication might be a common feature of ICEs relying for their mobility on a Mutator-like DDE transposase.

No three-dimensional structure of a Mutator-like transposase is presently available. Nevertheless, secondary structure predictions showed that, as with other DDE transposases, the five p-MULT families show an RNase H fold organization (supplementary fig. S2, Supplementary Material online). This analysis also revealed features specifically shared between the eukaryotic Mutator-like transposases and their prokaryotic counterparts, such as a conserved C/D(2)H signature a few amino acids after the second aspartyl residue of the catalytic triad and a long α-helical insert between the second aspartyl and the glutamyl residues (fig. 5). Shared functional features further underscore the relationships between eukaryotic and prokaryotic Mutator-like transposases. Interestingly, as for IS*256*, Tn*GBS*, and *Mycoplasma* ICE, circular forms have been observed in eukaryotic MULE, like Mu1 and Mu1.7 of the maize (Sundaresan and Freeling 1987) and in the α3 MULE of the yeast *Kluyveromyces lactis* (Barsoum et al. 2010). Thus, this mode of transposition might be a unifying feature of the Mutator superfamily of transposases.

The dramatic increase of genomic data provides opportunities to decipher the insertion specificity of transposable elements by comparing multiple insertion sites. We have characterized the diversity of insertion specificity among the five p-MULT families. The insertions of IS*256* (p-MULT 1) and of *Mycoplasma* ICE (p-MULT 5) appeared to be random. We have previously shown that Tn*GBS* ICEs insert specifically upstream σ_A_ promoters in a conserved orientation. We show here that this property is shared by both the IS*Lre*2 family encoding p-MULT 3 transposases and several members of the IS*Kra*4 group (p-MULT 4 family). By comparing transposase sequences from these two lineages, we identified a conserved motif predicted to be involved in this atypical insertion specificity among prokaryotic TEs (fig. 8). By analogy with the integrase of the yeast retrotransposon Ty3, which interacts with the transcription factors TFIIIB and TFIIIC (Kirchner et al. 1995), we proposed that the Tn*GBS* transposase interacts with a subunit of the RNA polymerase initiation complex (Brochet et al. 2009). The location of this motif just upstream the domain N2 interacting with the two IRs is compatible with such a model. It has been suggested that transposase-mediated circularization of IS*256* preferentially starts with a sequence-specific first-strand cleavage at the left IS terminus (Hennig and Ziebuhr 2010). Similarly, the asymmetric transposition of IS*911* was shown to be a result of differential recognition of IRr and IRl by the transposase (Rousseau et al. 2008). Based on the analysis of the orientation of insertion of the different TEs that target promoter regions, we propose that the asymmetric recognition of the two IRs is responsible for the specific orientation of most TEs encoding p-MULT 3 transposases with the IRr next to the targeted promoter sequence.

All the retrieved members of the IS*H6* (p-MULT 2) family except one were identified in archaea. Promoters in archaea are more similar to eukaryotic Pol II dependent promoters with an AT-rich TATA box-like element (Palmer and Daniels 1995). Interestingly, IS*H6* preferentially targets an AT-rich motif (AANATNTT) that is duplicated upon transposition (supplementary table S2 and fig. S1, Supplementary Material online). Thus, this insertion specificity might also lead to a preferential insertion of these ISs in promoter or intergenic regions. Interestingly, Pack-MULEs that are nonautonomous MULEs carrying fragments of cellular genes have been shown to preferentially insert into the 5′ end of genes (Jiang et al. 2011). Therefore, targeting promoter regions and avoiding transposition into genes seems to be a shared strategy among Mutator-like elements to limit the fitness cost on the host cell.

In conclusion, transposable elements encoding Mutator-like transposases are much more widespread and diverse in prokaryotes than previously thought. As in eukaryotes, they represent one major superfamily of transposable elements in prokaryotes. The late discovery of the expansion of this group was probably the result of the low protein sequence conservation that was only revealed by using an extensive cascade PSI-Blast search. The comparative analysis of these elements showed both unifying features in terms of the predicted structure and transposition mechanism, but also differences in terms of insertion specificity and of organization.

## Supplementary Material

Supplementary figures S1 and S2 and tables S1 and S2 are available at *Genome Biology and Evolution* online (http://www.gbe.oxfordjournals.org/).

Supplementary Data
