# Supplementary material for: The Diversity of Prokaryotic DDE Transposases of the Mutator Superfamily, Insertion Specificity, and Association with Conjugation Machineries
Source: Genome Biol Evol. 2014 Jan 13;6(2):260–72. doi: 10.1093/gbe/evu010 (PMC3942029; doi:10.1093/gbe/evu010)
Supplement: Supplementary Data [file supp_6_2_260__index.html]

The Diversity of Prokaryotic DDE Transposases of the Mutator Superfamily, Insertion Specificity, and Association with Conjugation Machineries — Supplementary Data 

# The Diversity of Prokaryotic DDE Transposases of the Mutator Superfamily, Insertion Specificity, and Association with Conjugation Machineries

## Supplementary Data

files

**Files in this Data Supplement:**

- Supplementary Data - pdf file
- Supplementary Data - xls file
- Supplementary Data - xls file
