# Supplementary material for: The Diversity of Prokaryotic DDE Transposases of the Mutator Superfamily, Insertion Specificity, and Association with Conjugation Machineries
Source: Genome Biol Evol. 2014 Jan 13;6(2):260–72. doi: 10.1093/gbe/evu010 (PMC3942029; doi:10.1093/gbe/evu010)
Supplement: Supplementary Data [file supp_evu010_Guerillot_et_al_Sup_mat.pdf]

Supplementary table and figure legends.

Table S1. Transposable elements encoding a transposase of the p-MULT superfamily.

The 731 non-identical proteins were retrieved by the cascade PSI-BLAST search. The p-MULT family is indicated in column A. When identified, the name of the corresponding TE is indicated in column B in accordance with the ISfinder database annotation scheme. The TE family name according to figure 2 is indicated in column C. The Genebank accession numbers of the transposase sequence and of the contig encoding it are indicated in columns D and E, respectively. Species and strain names, if known, are indicated in column F. The total number of IR-genome junctions retrieved and the number of IR-genome junctions with a predicted promoter at a distance of 14 to 18 bp from the IR ends are indicated in column G and H, respectively. The ratio of the number of junctions with a promoter to the total number of junctions is indicated in column I. When identified, the IRs length, the number of matches between IR-right and IR-left and their percentage of complementarity are indicated in columns N, O and P, respectively. The DR length and their sequence are in columns Q and R, respectively. Additional ORFs that do not code for the transposase and -1 frameshift in the transposase gene are indicated in columns T and U, respectively. In column V, the four transposases with an HTH_23 DNA-binding motif are indicated. The GeneBank transposase annotation is indicated in column W. The taxonomy of the species in which the transposase was detected is indicated in column X. Finally, the length of the identified TE, its sequence, the strand and the coordinates relative to the contig indicated in column E are described in the columns Y, Z (and AA for sequences larger than 32767 nucleotides), AB and AC-AD, respectively.

Table S2. Transposable elements insertion sites and σ_A promoter predictions.

For each IR-junction extracted from an identified TE, the p-MULT family of the transposase and the TE family names according to figure 2 are indicated in column A and B, respectively. IR-right or IR-left – genome junction is indicated in column C. The Genebank accession number of the transposase sequence is indicated in column D. The accession number of the contig and the coordinate of the insertion site are indicated in columns H and F, respectively. The core genome sequence next

to the IR is represented in column I. When a σ_A promoter was predicted by the PPP software at a distance from 14 to 18 bp, the promoter type according to PPP is indicated in column J. The distance between the IR and the putative -35 box of the σ_A promoter is indicated in column K. The -35 (TTGACA) and the -10 (TATAAT) box predicted are in column M and O. The E-value of the promoter prediction according to the PPP software (http://bioinformatics.biol.rug.nl/websoftware/ppp) is indicated in column Q. All extracted insertion sites are shown in the first Excel sheet, while only the non-identical insertion site that where used for the analysis depicted in figure 4 are shown in the second Excel sheet.

Figure S1. Inverted repeats of p-MULT encoding TEs.
(A) Sequence logos of the unaligned first 40 bp (IR-left) and last 40 bp (IR-right) of the five families of TEs encoding p-MULT transposases. (B) Sequence logo of the 8 bp sequence duplicated upon the insertion of IS*H6* related IS. Logos were generated by using WebLogo (Crooks, et al. 2004).

Figure S2. Secondary structure predictions of the five p-MULT families.
Secondary structure predictions were performed using the Jpred3 server (Cole, et al. 2008) and the transposase sequences YP_005745331 (IS*256*), NP_279987 (ISH6), NP_735564 (Tn*GBS2*), ZP_06972267 and YP_003515569 as query for p-MULT 1 to 5, respectively, and visualized with the Jalview software (Waterhouse, et al. 2009). Green arrows and red bars represent beta sheets and alpha helices, respectively. The most conserved residues in the protein sequence alignment are indicated. (A) The predicted secondary structure of the catalytic domain of each p-MULT family. Catalytic DDE residues are represented in red and the D/C(2)H motif in blue. The typical RNase H fold β1-β2-β3-α1-β4-α2/3-β5-α4-α5 and the predicted long alpha insert are indicated below the protein sequences. (B) The predicted secondary structure of the N-terminal region encompassing domain N1 and N2 of the five p-MULT families. For p-MULT 1 to 4, the conserved domain predicted to be involved in IR binding is shadowed.

References

Cole C, Barber JD, Barton GJ 2008. The Jpred 3 secondary structure prediction server. Nucleic Acids Research 36: W197-201.

Crooks GE, Hon G, Chandonia JM, Brenner SE 2004. WebLogo: a sequence logo generator. Genome Res 14: 1188-1190.

Waterhouse AM, Procter JB, Martin DM, Clamp M, Barton GJ 2009. Jalview Version 2--a multiple sequence alignment editor and analysis workbench. Bioinformatics 25: 1189-1191.

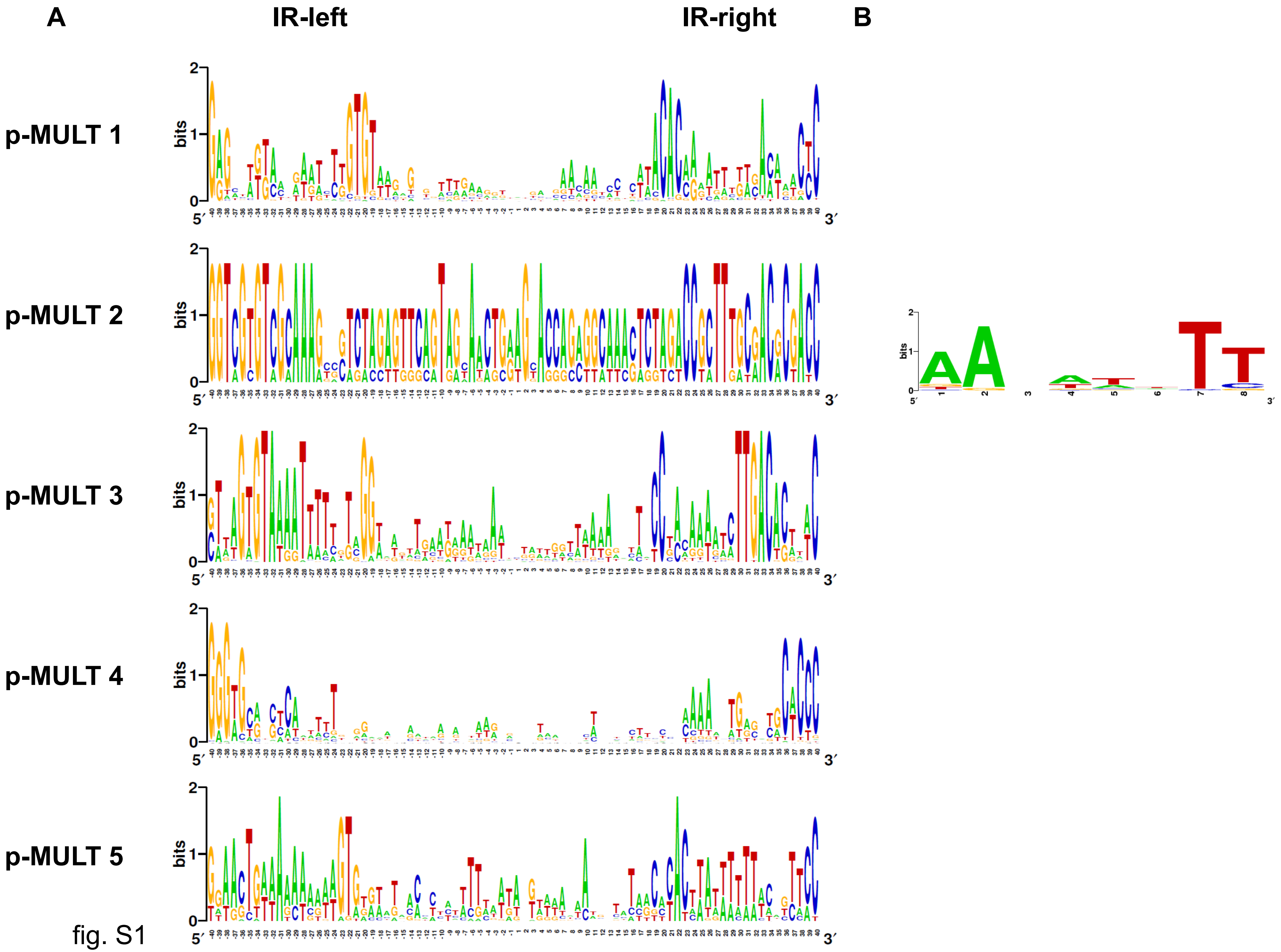
A
IR-left
IR-right
B
p-MULT 1
p-MULT 2
p-MULT 3
p-MULT 4
p-MULT 5
bits
5′
3′

fig. S1

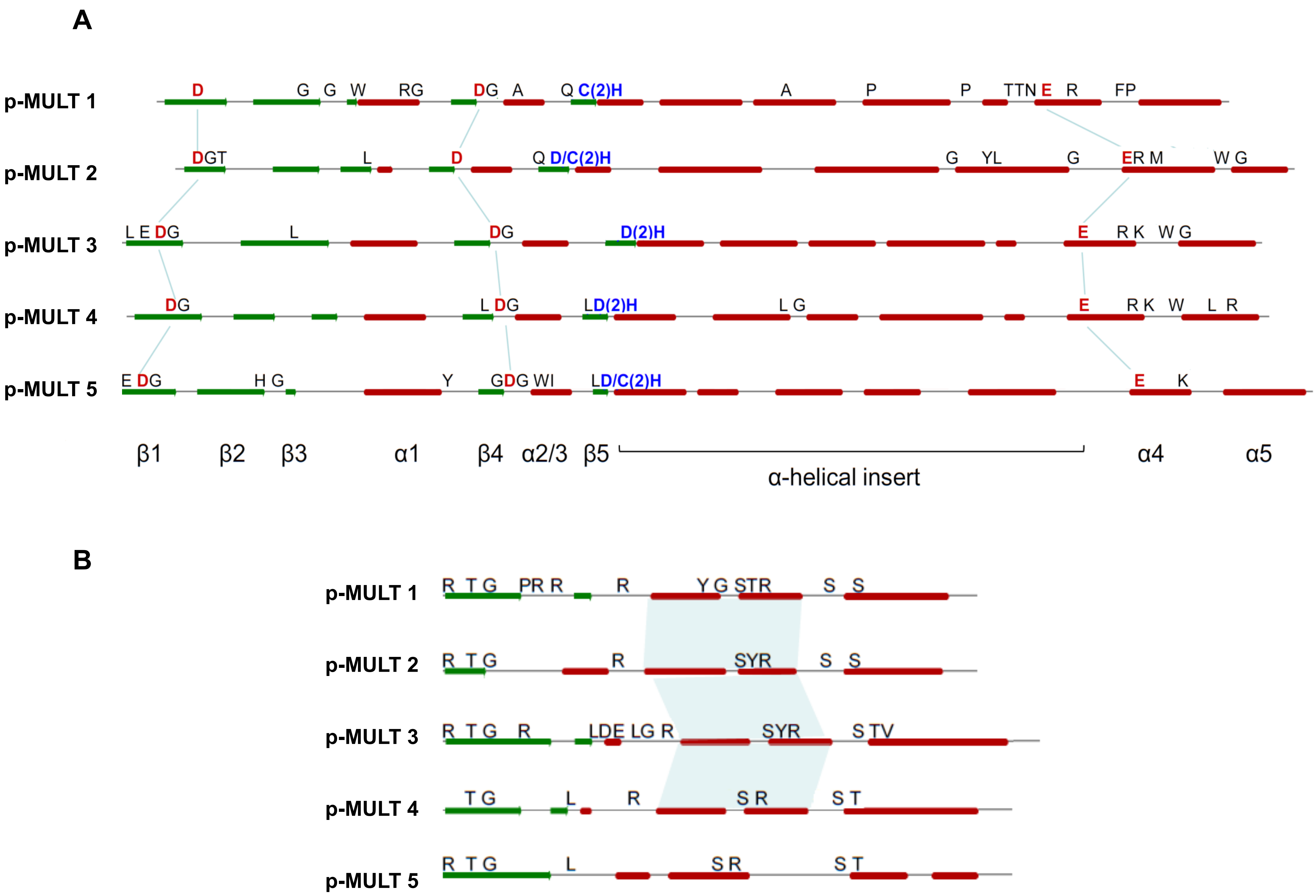
A
p-MULT 1
p-MULT 2
p-MULT 3
p-MULT 4
p-MULT 5
β1
β2
β3
α1
β4
α2/3
β5
α-helical insert
α4
α5
B
p-MULT 1
p-MULT 2
p-MULT 3
p-MULT 4
p-MULT 5

fig. S2
